# Improving sport horse selection using machine learning: Early prediction of elite-level show jumping performance

**DOI:** 10.1371/journal.pone.0357799

**Published:** 2026-09-15

**Authors:** Marco Zanchi, Clara Bordin, Michela Ablondi, Vittoria Asti, Andrea Summer, Emanuela Valle, Laura Ozella

**Affiliations:** 1 Department of Veterinary Sciences, University of Turin, Grugliasco, Italy; 2 Department of Veterinary Science, University of Parma, Parma, Italy; Universidade Federal de Mato Grosso do Sul, BRAZIL

## Abstract

Early identification of sport potential in show jumping horses remains challenging because competition outcomes vary widely, and elite-level performance develops after considerable time. In this study, we evaluated the applicability of machine learning approaches to competition records from horses identified through FEI participation, complemented by national-level records from Sweden, Belgium and France, with the aim of identifying horses likely to reach elite performance. Performance data from Fédération Équestre Internationale (FEI) competitions were analysed using an Extreme Gradient Boosting (XGBoost) classifier trained on longitudinal competition histories recorded before horses reached ten years of age. Animals were classified as champions if they achieved at least one clear round at 160 cm, while non-champions were defined as those that completed their competitive career without reaching this level. Model performance was assessed using nested cross-validation and compared with two single-rule-based classifiers based on maximum obstacle height thresholds. XGBoost outperformed baseline approaches across all evaluation metrics, achieving an average area under the ROC curve of 83.5% and an average precision of 80.0%. Analysis of probability trajectories showed that predictive information accumulates progressively over a horse’s career, with increasing separation between champion and non-champion predictions as competition history expands. Model performance improved substantially from approximately seven years of age onward. Permutation importance and SHapley Additive exPlanations (SHAP) analyses revealed that obstacle difficulty, performance consistency and age-related progression were among the most influential predictors. Overall, these findings demonstrate that interpretable machine learning models can extract meaningful predictive signals from early competition trajectories and support evidence-based talent identification in show jumping, while maintaining attention to performance development and horse welfare.

## Introduction

Show jumping is a major international equestrian discipline in which horses are progressively challenged to jump obstacles of increasing height and technical complexity without knocking them down (i.e., achieving a ‘clear round’), culminating in elite-level competitions at the highest fence heights [1,2]. This progressive structure poses a considerable challenge for breeders, trainers and owners, as elite-level performance can only be achieved and observed late in a horse’s career, while key breeding, training and career-orientation decisions must be made much earlier.

Elite-level show jumping performance (i.e., 160 cm classes) is generally achieved only at around nine or ten years of age [2,3], making early prediction of future performance particularly challenging. Traditionally, sport horse talent scouting relies primarily on human expertise [3], which may be affected by subjectivity and bias and thereby expose owners and breeders to potential economic losses, while also placing horses at risk of injury or compromised welfare, given the physical demands associated with high-level competition [4]. Over the past two decades, breeding associations (studbooks) have placed increasing emphasis on improving the selection of stallions and mares for breeding, with each studbook pursuing increasingly specialised horses for specific disciplines such as show jumping and dressage [5,6]. This trend reflects the increasing demand for horses that combine elite athletic performance with soundness and longevity, and the need to identify such individuals early in life, before substantial economic and training investments are made. Studbook selection is mainly based on genetic evaluations, which, depending on the breeding association, may incorporate competition results, performance tests (e.g., young horse competitions) and inspections (e.g., conformation, free movement) [1,7]. While studbook entry is typically based on pedigree and phenotypic assessments, genetic evaluations are primarily used to rank animals for breeding purposes rather than to predict the level of sport performance each horse will ultimately attain (e.g., amateur versus elite level). Accordingly, current breeding selection schemes may fail to identify horses with favourable breeding values that nevertheless have individual morphological or phenotypic traits limiting their suitability for high-level sport performance. Although several studbooks already incorporate morphological assessments into their selection processes, these evaluations are primarily used to define the estimated breeding value (EBV) rather than to predict the achievable sport performance level or the risk of injury. In such situations, horses would be more appropriately directed towards alternative careers, as competing at elite level may increase the risk of injury. From a breeding perspective, this difficulty in anticipating elite sport performance at an early age contributes to longer generation intervals and reduces breeding selection efficiency [7].

The use of machine learning algorithms, together with the increasing availability of computational power [8], may help address the growing demand for early prediction of performance and sport ability, thus improving the current sport selection strategy. Machine learning techniques are commonly used to perform classification with the aim of predicting a target variable (a process known as supervised learning) and forecasting performance represents one of its possible applications [9]. Unlike traditional statistical approaches, machine learning methods are well suited to handling complex, heterogeneous datasets and modelling non-linear relationships without requiring strong assumptions about underlying parametric distributions [10]. These advances have been widely adopted both in animal science, where machine learning is increasingly used to improve productivity and efficiency [10–13], and in human sports science, where it plays a key role in the early selection of promising athletes [14,15]. Despite the promising results obtained in livestock species and in human athletes, the application of these approaches to the equestrian sector is, to our knowledge, still extremely limited. Only a few studies have focused on horse racing [16–18], and these have largely followed research conducted on other racing sports (e.g., dog racing) [19]. Predictive, data-driven approaches remain largely unexplored in equestrian disciplines, particularly in show jumping.

Whether early competition records from international show jumping events contain sufficient predictive signal to identify future elite performers remains an open question. The aim of this study is to test whether an XGBoost classifier trained on competition records from horses identified through FEI participation, complemented by national-level records from Sweden, Belgium and France, can predict which horses will achieve elite-level performance in show jumping.

## Materials and methods

### Horse population and competition records

**Fig 1:**
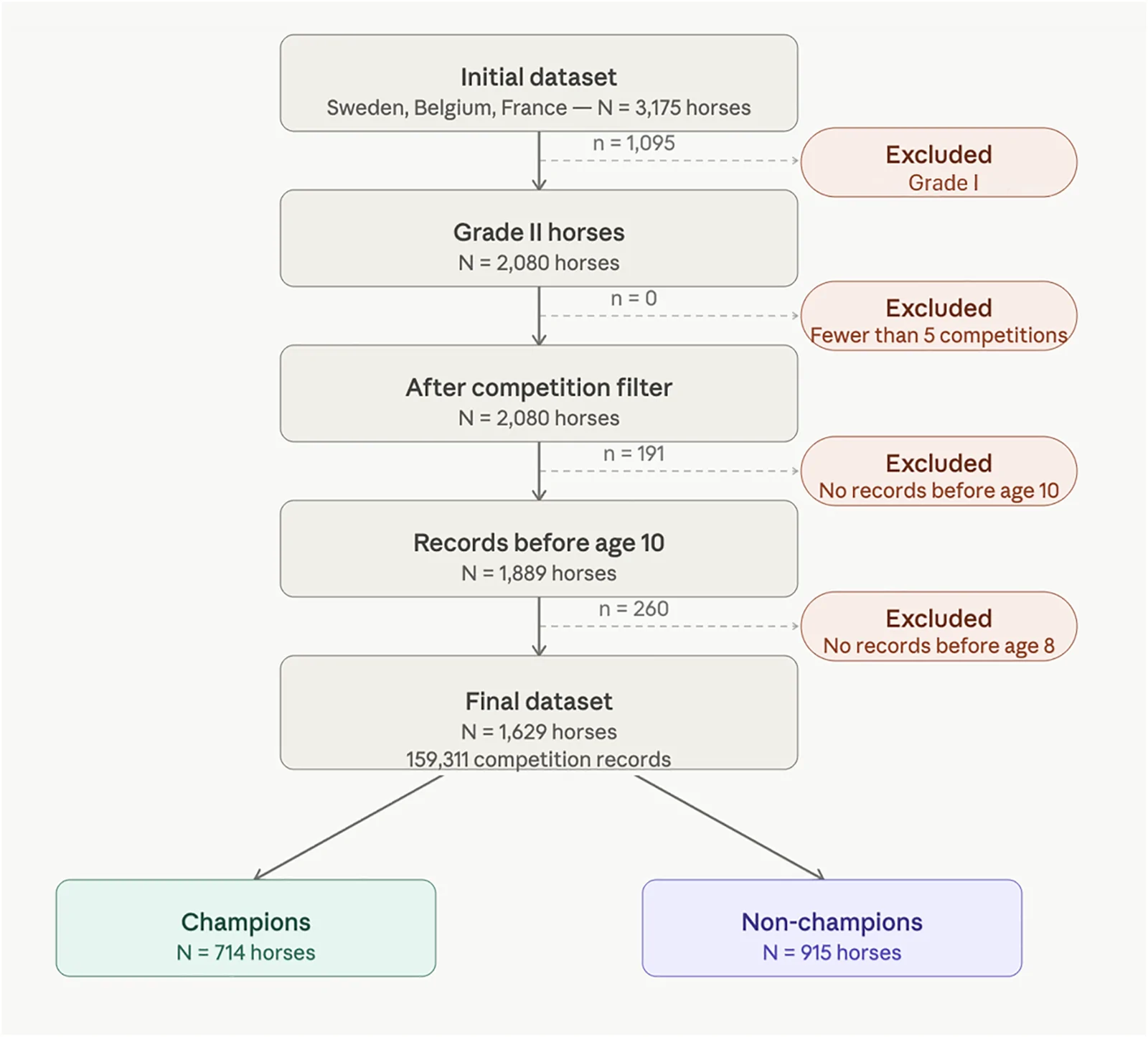
Flowchart of the data selection process.

**Fig 2:**
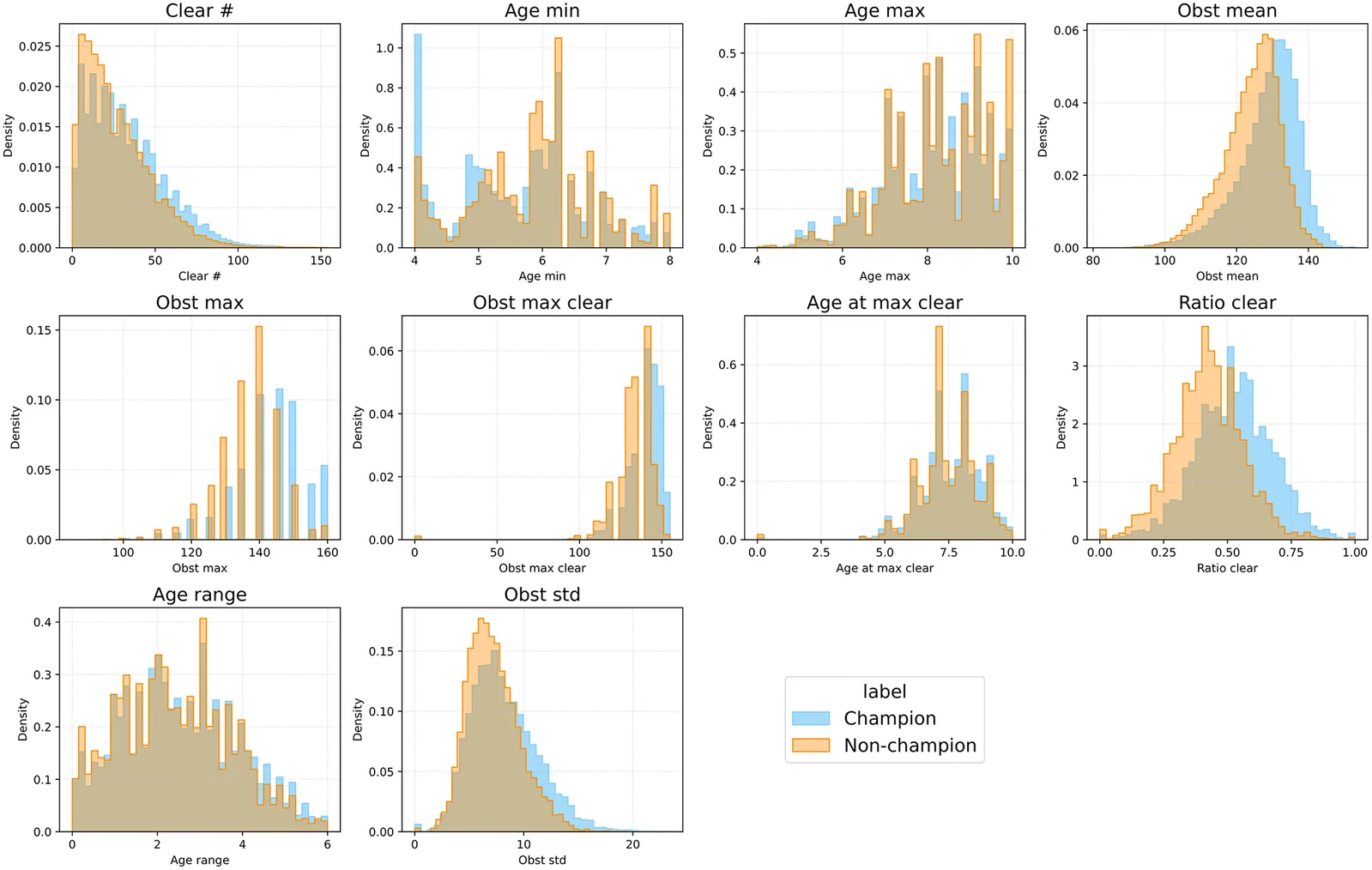
Feature distributions for champion vs. non-champion horses. Clear #: total number of clear rounds; Ratio clear: proportion of clear rounds over total attempts; Age min/max/range: minimum, maximum and range of competition age (years); Obst mean/max/std: mean, maximum and standard deviation of obstacle height (cm); Obst max clear: maximum obstacle height successfully cleared (cm); Age at max clear: age at which the highest obstacle was cleared (years). In the “Obst max clear” and “Age at max clear” panels, the peak at 0 does not represent a measured obstacle height or age: it corresponds to observation windows in which the horse had not yet achieved any clear round. These zeros are used as actual model inputs encoding the “no clear round yet” state (a distinct category rather than a measured value), consistent with the feature encoding described in the Methods; the co-occurring values of Clear # = 0 and Ratio clear = 0 identify the same windows.

We analysed competition records from horses identified through FEI participation, complemented by national-level records from Sweden, Belgium and France. The dataset is not limited to FEI-sanctioned events but includes the full competition record for each horse, comprising both international and national-level performances. This ensures that early career records (4–6 years of age), which typically take place at national level, are captured in the analysis. For each horse, competition-level information was available, including age at competition, obstacle height and clear round outcome. Age at competition was expressed as a decimal value in years, where the fractional part encodes the number of months elapsed since the most recent birthday (e.g., 7.5 years indicates a horse competing 6 months after its seventh birthday). To ensure sufficient performance history for model training, only horses with records from a minimum of five competitions were included in the analysis. The data selection process is summarised in Fig 1. The initial dataset comprised 3,175 horses, divided into two categories: Grade I, competing at levels below 140 cm; and Grade II, competing at or above 140 cm. Since the prediction task is directed at horses with the potential to reach top-level performance, the 1,095 Grade I horses were excluded, leaving 2,080 Grade II horses. We then required each horse to have at least five recorded competitions, the minimum we considered sufficient to characterise performance trajectories reliably. This filter did not remove any horse from the Grade II subset (n = 2,080 retained). The analysis was further restricted to horses with at least one record before 10 years of age (n = 191 excluded), and only horses with at least one record before the age of eight were retained (n = 260 excluded), yielding the final analytical sample of 1,629 horses corresponding to 159,311 competition records. This additional age-of-onset criterion was applied for modelling reasons: because features are computed from growing windows of at least five competitions occurring before ten years of age, horses whose first record appears only after eight years of age would contribute a single, temporally compressed observation window confined to the eight-to-ten-year interval. Such windows do not capture early-career development and yield degenerate age-related features, so retaining only horses with a record before eight years ensures an early and comparable observation window across the cohort. Because eight years is well above the modal age of competitive onset in the dataset (Fig 2), this criterion excludes only late-starting horses at the tail of the onset distribution. Within this cohort, horses that achieved at least one clear round in competitions held at 160 cm level, corresponding to the highest level of international show jumping, were classified as elite-level performers (“champions”; N = 714). This criterion was chosen to identify horses that reached elite-level competition, rather than to evaluate overall career success. Conversely, horses that did not reach this level and had competed until at least 17 years of age were classified as non-elite performers (“non-champions”; N = 915).

### Feature engineering

For each horse, only competition records occurring before 10 years of age were considered, as elite-level performance is typically achieved around this age [20]. This cutoff ensures that the large majority of horses have had sufficient opportunity to demonstrate their competitive potential, while restricting the analysis to early career records. For horses classified as champions, observations were further restricted to competitions occurring strictly before the first clear round at an obstacle height of 160 cm, to prevent information leakage (i.e., the inadvertent use of future information during model training, which would lead to overly optimistic performance estimates) and ensure a fair predictive setting. Each horse’s competition history was treated as a time-ordered sequence of events, with each event characterised by the horse’s age at competition, the obstacle height attempted and a binary indicator of clear-round performance. To generate fixed-length input features suitable for supervised learning (i.e., a fixed number of summary statistics describing each horse’s performance history), a growing-window approach was applied, whereby performance summaries were progressively computed over an increasing number of competitions, starting from a minimum of five events. Because feature construction is restricted to records occurring before ten years of age and each growing window must contain at least five events, ten horses that reached their fifth competition only after ten years of age retained fewer than five records within the pre-ten-year window and therefore contributed no windows to the feature matrix. Accordingly, while the analytical sample defined by the inclusion criteria comprises 1,629 horses (Fig 1), the effective model-training cohort comprises 1,619 horses.

For each observation window, summary features were extracted to describe the temporal evolution of performance. These features captured complementary aspects of competitive development and included indicators of performance consistency (e.g., number and proportion of clear rounds), age-related dynamics (e.g., minimum and maximum competition age, age range), and obstacle-related characteristics (e.g., mean and maximum obstacle height attempted, variability in obstacle height, highest obstacle cleared and age at the highest clear). When an observation window contained no clear round, the features describing the highest cleared obstacle and the age at the highest clear were set to 0. This encoding is a modelling choice rather than a plotting convention: because 0 lies below the range of observed obstacle heights and competition ages, XGBoost, which splits on the ordinal position of feature values, treats it as a distinct sentinel that isolates windows without any clear round while preserving the ordering among genuine values. The co-occurring values of the number and proportion of clear rounds (both equal to 0) provide an explicit indicator of this state. A formal description of the feature construction procedure is provided in **S1 File**.

#### XGBoost training procedure.

XGBoost (Extreme Gradient Boosting) is a tree-based ensemble learning method widely applied in structured data classification problems [21]. It builds a strong classifier by sequentially combining multiple weak learners (decision trees), where each tree attempts to correct the residual errors of the previous ones. Model training is driven by the minimization of a regularized objective function, combining a logistic loss term for binary classification with a regularization term that penalizes model complexity, making XGBoost resistant to overfitting while maintaining high predictive accuracy. Model optimization is performed using a second-order Taylor approximation of the loss function, and tree construction follows a greedy split-finding algorithm that maximizes the expected reduction in the objective function. XGBoost was implemented using the *xgboost* library (version 3.2.0) in Python (version 3.14.3). To ensure a rigorous assessment of the model’s predictive performance and to avoid any information leakage between horses, we adopted a strict nested cross-validation strategy with group-aware data splitting. Since each horse contributes multiple time-window samples, standard cross-validation would lead to overly optimistic performance estimates if observations from the same horse appeared in both training and test folds. To prevent this, we used each horse’s unique FEI identification code as grouping variable so that the data from each horse was entirely contained either in the training or in the test set of each fold. The validation scheme consisted of an *outer cross-validation loop* for model evaluation and an *inner cross-validation loop* for model selection. Specifically, a 5-fold cross validation was used in the outer loop with group-wise separation by horse. Inside each outer training split, hyperparameter selection for XGBoost was performed using a 3-fold inner cross validation procedure. Model optimization was driven by the area under the precision-recall curve $\left(AUC_{PR}\right)$.

The training and validation procedure is summarized below. In each iteration of the outer loop, four folds were used for model selection (inner training and validation), while one outer fold was held out exclusively for final testing. No information from the outer test fold was used during model selection, training, or classification threshold tuning. The procedure within each outer fold was as follows:

1. **Hyperparameter optimization (inner loop):** A randomized search was performed over a predefined hyperparameter space using the whole training data from the outer cross validation (data from 4 folds), applying horse-level grouped splitting to prevent information leakage during hyperparameter tuning. The configuration maximizing $AUC_{PR}$ was selected for model training.
2. **Inner cross-validation predictions:** Using the best hyperparameters, we performed inner 3-fold cross-validation again to obtain out-of-fold probability predictions for all training samples. These predictions approximate how well the model generalizes on unseen data during training and were used only for threshold estimation.
3. **Data-driven threshold selection:** Since binary classification based on a fixed probability threshold of 0.5 is arbitrary, we computed an optimal classification threshold using the Youden index:

$$\begin{array}{c} \theta^{*}=\underset{\theta }{arg\;max}\left(\text{TPR}\left(\theta \right)-\text{FPR}\left(\theta \right)\right) \end{array}$$ (1)

where $\theta^{*}$ was computed solely from inner out-of-fold predictions to avoid bias.

1. **Final model training:** Using the selected hyperparameters, a final XGBoost classifier was trained on the full outer training fold. This model, together with the optimal threshold $\theta^{*}$, was then used for unbiased evaluation on the held-out outer test fold.
2. **Evaluation on unseen horses:** The trained model produced probability estimates for the horses in the outer test fold, which were converted into binary predictions using the threshold $\theta^{*}$ derived from the inner loop.

To investigate how early in a horse’s career reliable predictions can be made, we performed an additional analysis in which the input data were restricted to competitions occurring before a given age threshold, ranging from 5 to 10 years in steps of one year. For each threshold, the model was retrained and evaluated using the same nested cross-validation procedure described above. This analysis quantifies how predictive information accumulates with age and helps identify the earliest stage at which accurate prediction of elite potential becomes feasible.

#### XGBoost evaluation metrics.

Model performance was evaluated on the external test folds of the nested cross-validation using both threshold-dependent and threshold-independent metrics. Predicted probabilities were generated for each sample, and binary predictions were obtained by applying the optimal classification threshold derived from the inner cross-validation loop. Threshold-dependent metrics included accuracy (ACC), sensitivity (SEN; true positive rate) and specificity (SPEC), computed from the confusion matrix. Sensitivity reflects the ability of the model to correctly identify champion horses, whereas specificity reflects its ability to correctly exclude non-champions. Threshold-independent performance was assessed using the area under the receiver operating characteristic curve (AUC) and average precision (AP), which approximates the area under the precision–recall curve and is particularly informative in the presence of class imbalance. Performance was computed independently for each outer fold, and final values were reported as the mean across folds. All evaluation metrics were computed at the window level. Because each horse contributes multiple growing-window observations, horses with longer pre-ten-year competition histories contribute more observations to the computed metrics. We report window-level performance because it reflects the intended operational use of the model, in which a prediction is produced at each stage of a horse’s early career as competition history accumulates, rather than as a single retrospective judgement per horse; evaluating across all windows therefore characterizes the model as it would actually be applied. The group-aware nested cross-validation ensures that all windows of a given horse are assigned entirely to either the training or the test fold, so window-level evaluation does not introduce information leakage across horses. Definitions of the evaluation metrics and their mathematical formulation are provided in the **S2 File**.

#### Single-rule-based classifiers: Maximum obstacle threshold.

To assess whether machine learning provides an improvement over simple domain heuristics, we compared XGBoost with two single-rule-based classifiers derived from a general assumption in show jumping: *horses that reach higher obstacle heights are more likely to become champions*. The first classifier (Baseline 1) uses the maximum obstacle height attempted within the competition history, including unsuccessful attempts. The second classifier (Baseline 2) uses only the maximum obstacle height successfully cleared (clear rounds only). Accordingly, we constructed a baseline classifier that predicts championship potential by thresholding the maximum obstacle height achieved within the time windows of the competition history.

For each horse h, let $\{o_{h,\text{1}},o_{h,\text{2}},\ldots ,o_{h,L}\}\;$denote the sequence of obstacle heights attempted in the first L competitions (L ranges from 5 to the maximum records length). The baseline score is defined as:


$$\begin{array}{c} s_{h}(L)=\underset{\text{1}\leq t\leq L}{\text{max}}o_{h,t} \end{array}$$ (2)

It was also computed using only successful jumps (clearRound=1), yielding a stricter variant:


$$\begin{array}{c} s_{h}^{\text{clear}}(L)=\underset{\text{1}\leq t\leq L}{\text{max}}\{o_{h,t}|{\text{clearRound}}_{h,t}=\text{1}\} \end{array}$$ (3)

A horse is classified as champion if this score exceeds a threshold O:


$$\begin{array}{c} {\hat{y}}_{h}=I\left(s_{h}\left(L\right)\geq O\right) \end{array}$$ (4)

We tuned O in a data-driven manner within a nested cross-validation framework matching the evaluation setup of XGBoost. For each outer training fold, we scanned a discrete threshold grid O∈{120,121,…,160}cm, choosing $O^{*}$ as the value maximizing the F1 on the training fold. The best threshold $O^{*}$ was then used to compute test set metrics per outer fold using the same evaluation criteria as XGBoost (i.e., AUC, AP, ACC, SEN and SPEC) ensuring a fair comparison. We also evaluated a stricter version of the baseline in which only clear rounds were used to define $s_{h}(L)$, i.e., ignoring obstacle heights from unsuccessful attempts.

#### XGBoost interpretation: Permutation importance.

To evaluate the contribution of individual features to the predictive performance of the XGBoost model, permutation importance (PI) was computed.Permutation importance assesses feature relevance by quantifying the decrease in model performance observed when the relationship between a given feature and the target variable is disrupted through random permutation. For each fold of the outer cross-validation, permutation importance was computed on the external test set to ensure an unbiased interpretation of the model. Given a trained XGBoost model f and a test feature matrix X, the importance of feature j is estimated by measuring the variation in the evaluation metric M (AUC in our case) after randomly permuting the values of feature j across the test samples:


$$\begin{array}{c} \text{PI}(j)=M(f,X)-M\left(f,X_{\pi (j)}\right) \end{array}$$ (5)

where $X_{\pi \left(j\right)}$ denotes the feature matrix in which only the j−th column has been permuted. A higher value of PI(j) indicates a stronger contribution of the feature to the predictive ability of the model. To enhance robustness, permutation importance was computed using n=30 random permutations per feature and subsequently averaged across all outer folds. Importance values were aggregated across folds by computing the mean and standard deviation for each feature. This analysis allowed us to identify which performance descriptors most strongly contributed to early prediction of championship potential.

#### XGBoost interpretation: SHAP analysis.

To interpret the predictions of the XGBoost model, we computed Shapley Additive Explanations (SHAP) [22,23] on the external test set of each outer cross-validation fold. SHAP is an interpretability framework that decomposes a model prediction into additive contributions of individual features, thereby quantifying how each feature increases or decreases the predicted probability for a given observation. In our implementation SHAP values were computed on the raw margin (log-odds) output of the model, so that each value quantifies the additive contribution of a feature to the model’s log-odds output rather than to the predicted probability. Because the logistic link relating log-odds to probability is monotonic, a positive SHAP value still indicates that a feature increases the predicted probability of belonging to the positive class (champion) and a negative value indicates a decrease, whereas the magnitude of a SHAP value corresponds to a change in log-odds and not to a direct change in probability. SHAP values were computed independently for each outer fold and subsequently aggregated across folds to obtain a global interpretation of feature effects.

## Results

### Feature distributions in champion and non-champion horses

To describe the empirical differences between champion and non-champion horses, we analysed the distributions of the input features used by the model (Fig 2).

Clear differences between the two groups were observed for several obstacle-related features. Champion horses showed higher values of mean obstacle height (Obst mean), maximum obstacle height attempted (Obst max), and particularly maximum obstacle height cleared (Obst max clear). Champion horses also exhibited a higher proportion of clear rounds (Ratio clear). The distribution of obstacle height variability (Obst std) was shifted towards higher values in champion horses, indicating a broader range of attempted obstacle heights. Age-related features showed largely overlapping distributions between the two groups, with the exception of minimum competition age (Age min). Distributions of age range, maximum competition age (Age max), and age at maximum cleared obstacle (Age at max clear) were similar for champions and non-champions. In contrast, the distribution of minimum competition age (Age min) differed between groups, with champion horses showing a higher frequency of earlier competition onset.

### Predictive performance of XGBoost and baseline models

The average predictive performance across the external cross-validation folds, comparing XGBoost with two single-rule-based classifiers based on maximum obstacle height heuristics, is reported in Table 1.

**Table 1:** Evaluation metrics. The table shows the average evaluation metrics for XGBoost and two single-rule-based classifiers across the outer folds of the nested cross-validation. Baseline 1: single-rule-based classifier using the maximum obstacle height attempted (including unsuccessful attempts). Baseline 2: single-rule-based classifier using the maximum obstacle height successfully cleared (clear rounds only). AP: average precision; AUC: area under the ROC curve; ACC: accuracy; SEN: sensitivity; SPEC: specificity.

Model	AP	AUC	ACC	SEN	SPEC
XGBoost	80.0%	83.5%	74.9%	75.5%	74.5%
Baseline1	62.6%	68.3%	59.8%	75.7%	47.1%
Baseline2	64.0%	69.8%	64.1%	69.8%	59.6%

Across all evaluation metrics, XGBoost achieved higher performance than both baseline models. In particular, XGBoost showed higher values of AUC (83.5%) and average precision (80.0%), as well as higher accuracy (74.9%) and specificity (74.5%), compared with both baseline approaches. Sensitivity was comparable between XGBoost and Baseline 1, whereas specificity was substantially lower for both baseline models.

### Temporal evolution of predicted probabilities across competition history

**Fig 3:**
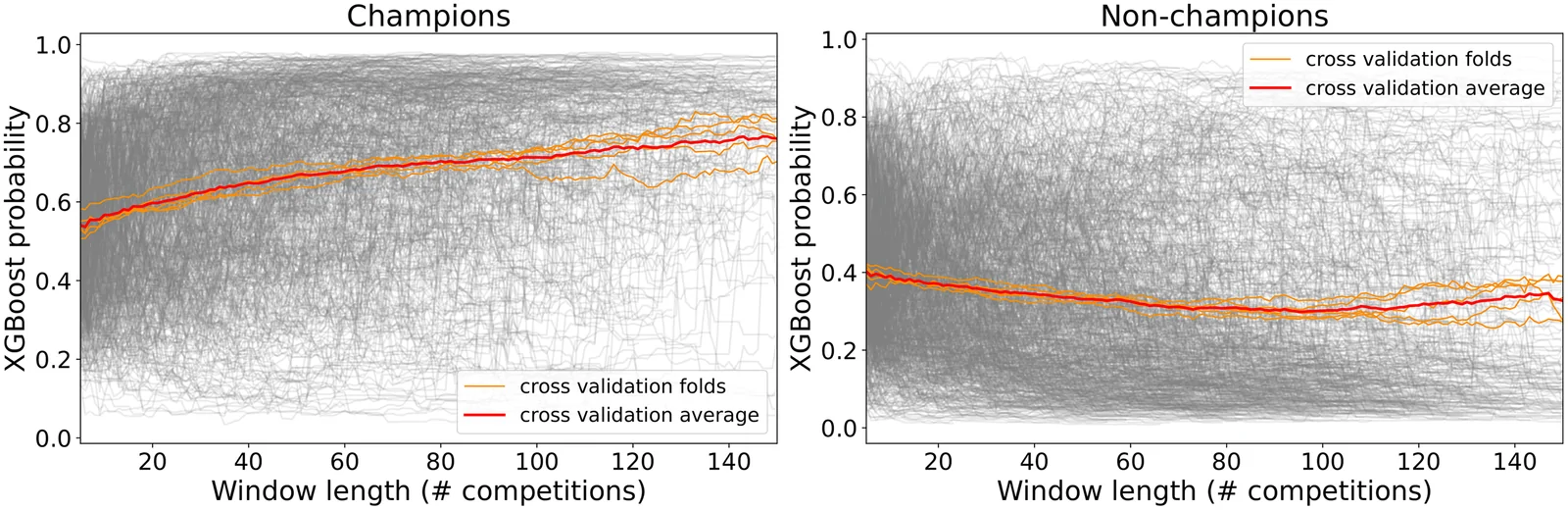
Predicted probability trajectories for champion (left panel) and non-champion (right panel) horses. Each grey line represents the probability trajectory of a single horse across increasing window lengths (i.e., as more competitions are included in the prediction window). Orange lines represent fold-level averages from the outer cross-validation folds, and the red line represents the overall cross-validation average across all folds. Champions are defined as horses that achieved at least one clear round at an obstacle height of 160 cm. Non-champions are horses that completed their competitive career without reaching this level.

To further investigate how predictive information accumulates over time, we analysed the evolution of the predicted probabilities produced by XGBoost at different stages of each horse’s competitive career. Because each horse contributes a sequence of competition results, XGBoost produces a *probability trajectory* that reflects how the estimated likelihood of becoming a champion evolves as more competitions are observed. Fig 3 shows the probability trajectories for champion (left panel) and non-champion (right panel) horses.

### Model performance as a function of age

**Fig 4:**
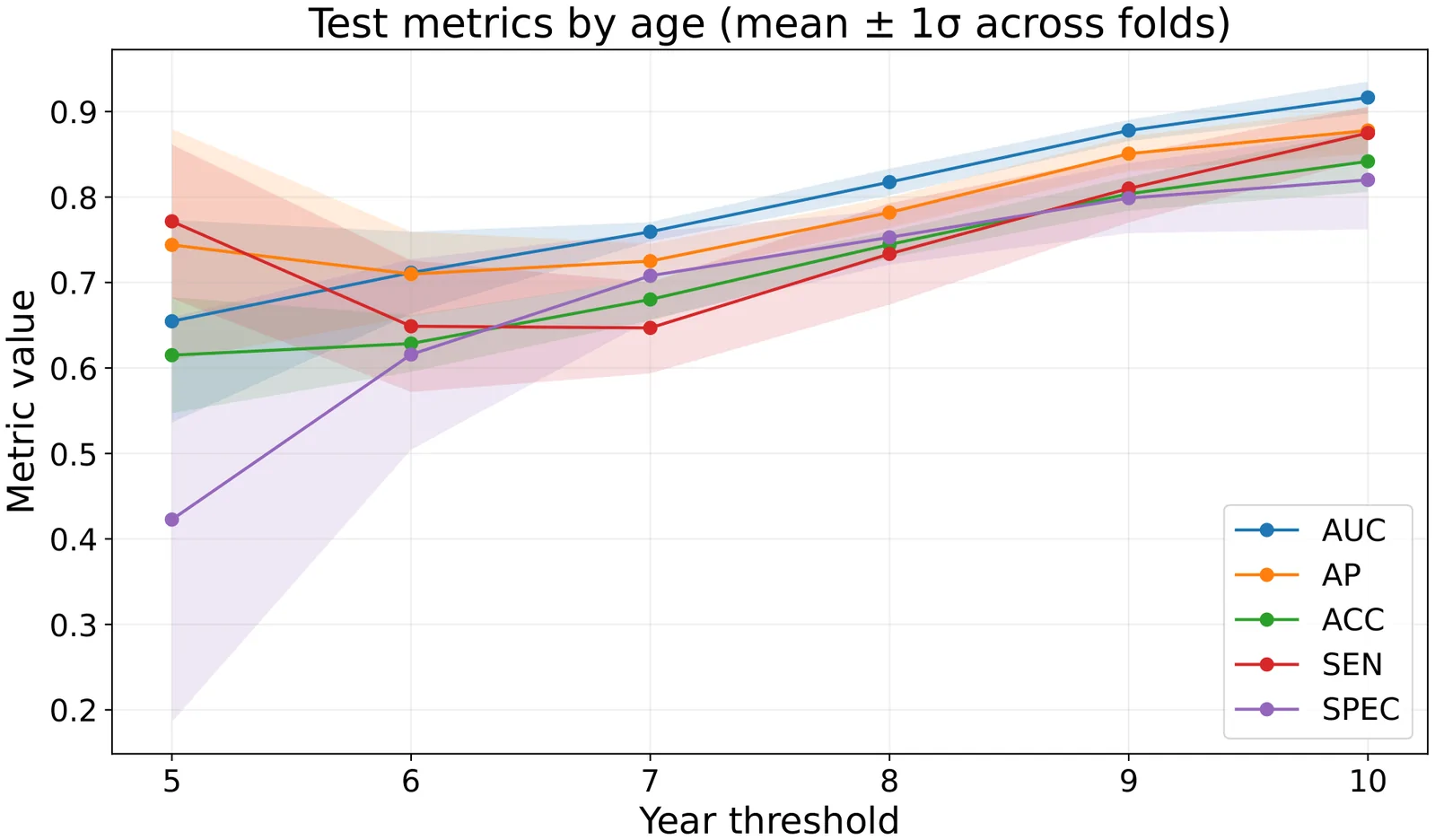
Test metrics at different horse ages. Values of AUC (area under the ROC curve), AP (average precision), ACC (accuracy), SEN (sensitivity) and SPEC (specificity) are shown for age thresholds ranging from 5 to 10 years. Each point represents the mean across the outer folds of the nested cross-validation, while the shaded regions indicate one standard deviation, reflecting the variability of predictive performance across validation splits.

We also investigated how early in a horse’s career reliable predictions can be made by restricting the input data to competitions occurring before increasing age thresholds and re-evaluating model performance using the same nested cross-validation procedure (Fig 4).

Most evaluation metrics (AUC, AP, ACC, SEN and SPEC) generally improved as higher age thresholds were considered, indicating higher predictive performance with increasing amounts of competition history. At younger ages (5–6 years), model performance, while above chance level, does not yet reach a level of operational reliability sufficient to support individual selection decisions. In particular, at 5 years of age specificity drops to approximately 0.40, resulting in a high false-positive rate. At 6 years, metrics improve to the 0.60–0.70 range, indicating a genuine but still limited discriminative ability. From 7 years of age onward, model performance improved substantially, with both AUC and average precision exceeding 0.70 on average. Performance continued to increase at higher age thresholds, with reduced variability across cross-validation folds.

#### Model interpretation.

**Fig 5:**
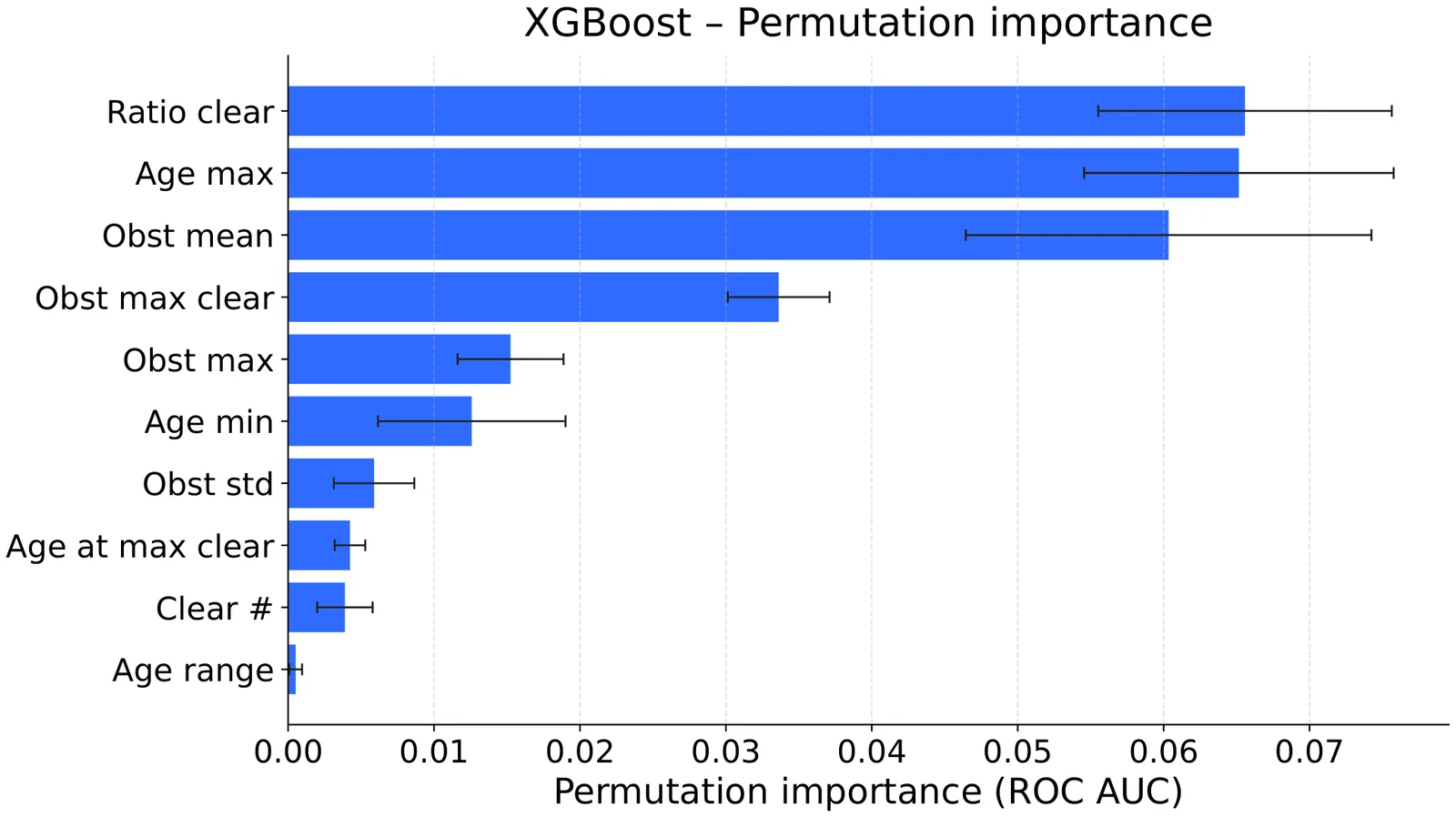
Permutation importance of XGBoost features. Bars show the mean decrease in ROC AUC when each feature is randomly permuted, averaged across outer folds of the nested cross-validation. Error bars indicate one standard deviation. Higher values correspond to greater importance in the model’s predictive ability. Feature abbreviations: Clear #, total number of clear rounds; Ratio clear, proportion of clear rounds over total attempts; Age min, Age max and Age range, minimum, maximum and range of competition age (years); Obst mean, Obst max and Obst std, mean, maximum and standard deviation of attempted obstacle height (cm); Obst max clear, maximum obstacle height successfully cleared (cm); Age at max clear, age at which the highest obstacle was cleared (years).

To characterize the contribution of individual input features to model predictions, we conducted a global feature importance analysis, using two methods: permutation importance and SHAP. Understanding which features drive model decisions is essential not only for assessing the transparency and reliability of the prediction process but also for identifying domain-relevant performance indicators that may act as early markers of sport potential. Fig 5 reports the average permutation importance computed across the outer folds of the nested cross-validation. This analysis measures the decrease in predictive performance (in terms of ROC AUC) when the values of a given feature are randomly permuted, thereby quantifying its contribution to the model’s discriminative ability.

**Fig 6:**
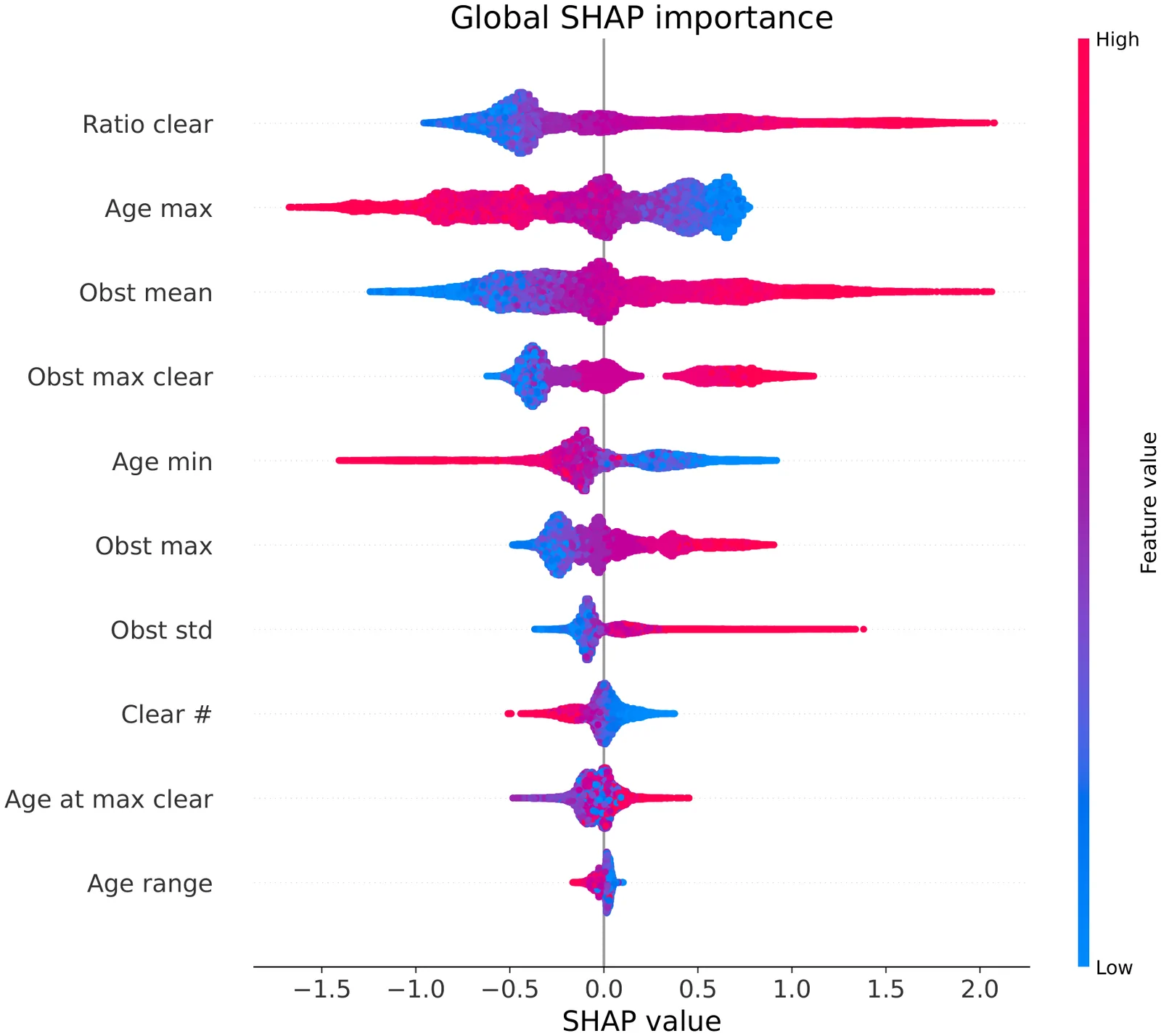
Global SHAP feature importance. Summary plot of SHAP values computed on the outer test folds of the nested cross-validation. SHAP values are expressed on the raw margin (log-odds) scale of the model (model_output = ’raw’); positive values increase, and negative values decrease, the predicted log-odds, and therefore the predicted probability, of being a champion, whereas their magnitude represents a change in log-odds rather than in probability. Each dot represents the SHAP value of a feature for one prediction; color indicates the corresponding feature value. Features are ordered by mean absolute SHAP value, showing their overall contribution to the model. Feature abbreviations: Clear #, total number of clear rounds; Ratio clear, proportion of clear rounds over total attempts; Age min, Age max and Age range, minimum, maximum and range of competition age (years); Obst mean, Obst max and Obst std, mean, maximum and standard deviation of attempted obstacle height (cm); Obst max clear, maximum obstacle height successfully cleared (cm); Age at max clear, age at which the highest obstacle was cleared (years).

Obstacle-related features showed the highest importance values, followed by age-related progression features, whereas other features exhibited lower contributions. Fig 6 shows the SHAP values computed across all outer test folds. Each point represents a single prediction for a given feature, with horizontal position indicating the magnitude and direction of the SHAP value on the log-odds scale (i.e., positive values increase, and negative values decrease, the model’s predicted log-odds, and therefore the predicted probability, of being a champion), and colour representing the feature value. Features are ordered by mean absolute SHAP value, reflecting their overall contribution to model predictions.

## Discussion

### Feature distributions and early performance patterns

The descriptive analysis of feature distributions provides an important complementary perspective to the machine learning results, allowing a direct comparison between champion and non-champion horses independently of model assumptions. The clear separation observed for variables related to obstacle height and clearance performance confirms that differences in competitive ability between the two groups are already visible at the descriptive level, supporting the validity of the predictive signal captured by XGBoost. As expected, champion horses consistently reached higher obstacle heights and displayed higher clearance ratios. More notably, the distribution of obstacle height variability was also shifted towards higher values in champion horses, indicating a broader range of attempted obstacle heights. These findings are in line with expert knowledge in show jumping, in which consistent performance at higher obstacle heights characterizes elite horses [1,2].

In contrast, most age-related progression features showed substantial overlap between champion and non-champion horses, indicating that age alone is a weak discriminator of future performance and must be considered in combination with other performance indicators. Nevertheless, the distribution of minimum competition age differed between the two groups with horses who eventually reached the champion level more frequently entering competition at earlier ages, in line with previous observations in show jumping populations [3]. In particular, our findings reveal that champion horses show a marked peak at four years of age, indicating an earlier entry into competitive activity compared with non-champions. This association is observational and may reflect several factors, including earlier identification of promising horses by owners and trainers, deliberate management strategies, or data availability, rather than a direct contribution of early competition exposure to future elite performance [3]. The earlier competitive onset observed in horses who eventually reached the champion level may also reflect deliberate management decisions by owners and trainers, who may expose promising horses to competition at a young age to assess their potential, subsequently withdrawing them for further development if results are encouraging. This suggests that early competition records may capture not only the horse’s intrinsic athletic potential but also the strategic decisions of the human team surrounding it. However, this should not be interpreted as encouragement for excessively early competitive involvement, as highly competitive demands at young ages may pose risks to musculoskeletal development and long-term welfare [4]. Instead, early exposure to competition may be viewed as an indicator of favourable developmental pathways supported by responsible training and individualised management.

### Predictive performance and comparison with baseline models

XGBoost was selected for this study because it is well suited to heterogeneous, non-linear data and does not require assumptions about the underlying distribution of input variables, making it particularly appropriate for modelling longitudinal competition performance records. Model performance was assessed using a combination of complementary evaluation metrics. The evaluation included both threshold-independent metrics (AUC and AP) and threshold-based ones (ACC, SEN, SPEC). The results clearly indicate that XGBoost outperforms both baseline approaches across all metrics. In particular, it achieves the highest Area Under the ROC Curve (AUC = 83.5%) and Average Precision (AP = 80.0%), demonstrating a superior ranking ability in distinguishing future champion horses from non-champions. This is a relevant improvement over the best-performing baseline (AP = 64%, AUC = 69.8%), showing that the model captures informative patterns in the competition history that go beyond simple performance thresholds. Because XGBoost was optimized using average precision (AUCPR) as the training objective, the model was trained to perform well under class imbalance, where champions represent the minority class. In this context, false positives correspond to horses incorrectly classified as future champions, which has practical implications: misclassifying a non-champion as a champion may induce breeders, trainers, or investors to allocate substantial training resources inefficiently. The model achieves relatively balanced sensitivity (75.5%) and specificity (74.5%), indicating that it is able to correctly identify a considerable proportion of champion horses while maintaining a controlled false positive rate. This trade-off is crucial for real-world applicability in horse performance prediction, where overestimating champion potential must be avoided.

### Temporal evolution of predicted probabilities

The probability trajectories reveal a clear temporal pattern in how predictive information accumulates over a horse’s competitive career. At early stages, when only a limited number of competitions is available, predicted probabilities remain close to 0.5, indicating limited discriminative power based on very limited performance history. However, as more competitions are observed, the predicted probabilities for future champion horses increase steadily, suggesting that the model progressively accumulates evidence about future champion potential. This behaviour confirms that performance signals relevant for elite success emerge gradually rather than abruptly, and that early competition dynamics carry predictive value.

In contrast, the probability trajectories of non-champion horses show an opposite pattern. Unlike the champions, the trajectories of non-champions display a declining trend. At early stages of the career, the model assigns an average predicted probability of approximately 0.4, reflecting initial uncertainty about the horse’s long-term potential due to limited data. However, as more competitions are observed, the predicted probability progressively decreases, indicating growing model confidence in classifying these horses as non-champions. This pattern suggests that non-champion horses tend to exhibit performance limitations that become gradually more evident through their competition history, leading the model to strengthen its negative predictions over time. When comparing the probability trajectories of champion and non-champion horses, the separation between the average trajectories becomes increasingly pronounced as more competitions are observed. This temporal divergence confirms that the model captures a gradual developmental trajectory, in which predictive signals accumulate progressively over time. The increasing separation between groups indicates that elite potential is reflected in a consistent pattern of performance evolution, not in sporadic exceptional results. In this sense, the model appears to capture talent as a dynamic and longitudinal process, where small but persistent performance differences compound across competitions and progressively shape future outcomes. This is consistent with the idea that elite success in show jumping may emerge through developmental continuity rather than abrupt breakthroughs, supporting the relevance of tracking performance trajectories instead of focusing on individual competition results.

The progressive accumulation of predictive information observed in our model is consistent with the broader sport science literature, which highlights the importance of longitudinal performance tracking for talent identification and development [8,24]. Rather than relying on single assessments, monitoring performance trajectories over time has been shown to provide more reliable indicators of future elite potential across different sports. It is worth noting that individual probability trajectories show considerable heterogeneity; some future champions receive relatively high predicted probabilities from early in their career, while others are identified only later. However, given that overall model performance at 5–6 years of age is only slightly above chance level (Fig 4), early high probabilities should be interpreted with caution, as they may reflect data noise rather than genuine predictive signal.

### Age-dependent emergence of predictive performance

The results show a consistent improvement across all evaluation metrics (AUC, AP, ACC, SEN and SPEC) as more competition history is accumulated. At very early ages (5–6 years), model performance is only slightly above chance level, indicating that competition results at the beginning of a horse’s career are still noisy indicators of long-term jumping potential. However, starting from approximately seven years of age, the model achieves substantially higher performance, with both AUC and average precision exceeding 0.70 on average. This indicates that by this stage, the performance trajectories of future champion and non-champion horses have diverged sufficiently for the model to make meaningful predictions. Although experienced riders and trainers may already have some intuition about a horse’s potential at this age, the present approach offers complementary advantages: it provides an objective and reproducible assessment applicable at scale across large populations, and captures complex longitudinal patterns that may not be immediately apparent even to expert observers. Furthermore, a reliable prediction at seven years still precedes the typical age of elite-level achievement by approximately three years, representing a meaningful window for breeding, training, and investment decisions. This finding aligns with equine sport research showing that competitive performance in show jumping improves progressively with age, with peak performance generally reached around ten years of age [25]. Consequently, age seven appears to represent a transition point at which competition performance begins to reflect stable athletic capability rather than early developmental variability. From a practical perspective, these results suggest that predictive screening for talent identification could be reliably initiated from around seven years of age, without incurring excessive uncertainty or false detections.

### Interpretability of predictive features

The combined use of permutation importance and SHAP analysis provided complementary insights into the factors driving the predictions of the XGBoost model, allowing both a global ranking of feature relevance and a detailed examination of how individual predictors influence the estimated probability of elite-level performance.

Our results highlight that obstacle-related features play a central role in prediction: in particular, the mean obstacle height attempted and the clear-round ratio are the two most influential predictors. This finding is partially expected, as champion status is defined by achieving a clear round at 160 cm. However, the model captures these features across the horse’s competitive history, indicating that it is not merely detecting the defining event itself, but rather identifying a consistent progression toward higher obstacle levels and sustained performance efficiency over time. These two variables capture complementary aspects of performance: while the mean obstacle height attempted reflects the technical difficulty faced by the horse, the clear-round ratio quantifies performance consistency and jumping accuracy over time. In addition, features related to age progression, such as maximum competition age, also showed notable importance, indicating that both competitive exposure and performance evolution contribute to characterizing championship potential [1]. Overall, these results indicate that the model captures a biologically and sport-relevant combination of ability, consistency and progression, in line with expert knowledge in show jumping horse evaluation.

In addition to permutation-based importance, SHAP values were used to provide a more fine-grained interpretation of how individual features contribute to model predictions. Whereas permutation importance quantifies the global relevance of each feature through its impact on model performance, SHAP allows each prediction to be decomposed into additive feature contributions, thereby providing insight into both the direction and magnitude of feature effects. Consistent with the permutation importance analysis, obstacle-related features and indicators of performance consistency emerged among the most influential predictors. In particular, the mean obstacle height attempted, the clear-round ratio, and maximum competition age ranked among the features with the highest overall contribution to model predictions.

Moreover, SHAP analysis provides additional insight into the structure of these effects. Higher obstacle-related values were systematically associated with positive SHAP contributions on the log-odds scale, corresponding to an increased predicted probability of achieving elite-level performance. Conversely, higher values of maximum competition age were predominantly associated with negative SHAP contributions, suggesting that horses continuing to compete for longer periods without reaching elite-level outcomes are more likely to be classified as non-champions. Together, these patterns indicate that the model captures dynamic performance trajectories rather than relying on isolated competition outcomes. These findings are consistent with recent machine learning studies in show jumping that have applied SHAP-based interpretability to FEI competition data. Hutchinson and Schwerha (2024) [26] analysed horse–rider dyad performance using CatBoost and SHAP, showing that cumulative experience and competition exposure were among the strongest predictors of ranking outcomes. Although that study focused on the performance of horse–rider combinations rather than on the horse alone, the convergence of results highlights the central role of longitudinal performance information across different modelling frameworks.

Overall, the agreement between permutation importance, SHAP analysis and recent machine learning studies indicates that the model captures a biologically and sport-relevant combination of ability, consistency and performance progression, in line with established criteria used for the evaluation of show jumping horses [1]. This convergence reinforces the interpretability and reliability of the predictive framework and supports the use of transparent machine learning approaches for evidence-based talent identification in show jumping. A relevant limitation of the present study is that the model relies exclusively on competition records and does not account for several environmental and management factors that are known to influence show jumping performance. Rider experience and training quality have been identified as key determinants of competitive success [25], and individual factors such as horse temperament and management conditions may further modulate performance trajectories [27]. The absence of such information in FEI competition records represents an inherent constraint of data-driven approaches based on publicly available databases. Future work integrating rider-level data, training history, and individual management information could substantially improve predictive accuracy and provide a more comprehensive picture of the factors driving elite performance in show jumping. Furthermore, the model treats competition records as an ordered sequence without accounting for the time intervals between consecutive events. Irregular competition schedules due to injury, rest periods, or management decisions may introduce noise in the performance trajectories. Additionally, horses with interrupted or prematurely terminated competitive careers (whether due to musculoskeletal injuries or the reproductive use of mares) were excluded from the analytical sample by design, as non-champion horses were required to have competed until at least 17 years of age. As a result, the model may not generalise to horses whose careers were curtailed before reaching this threshold, representing an important limitation for real-world applicability. Future work could address these limitations by integrating temporal information between competitions, injury records, and reproductive history, where available. In addition, the requirement that each horse have at least one competition record before eight years of age biases the analyzed sample towards earlier-starting horses and restricts the model’s applicability to horses that enter competition by approximately this age; horses beginning their competitive career later fall outside the population to which the present model applies.

## Conclusions

Our results demonstrate that machine learning can extract predictive signals from early competition trajectories that are not captured by simple single-rule-based classifiers, supporting the use of data-driven models in equine talent identification. The XGBoost model outperformed simple baseline approaches, with obstacle difficulty, clearance ratio, and performance progression over time emerging as the most informative features. Predictive accuracy improved notably from around seven years of age, when performance becomes more consistent and reliable. While competition history from earlier career stages (i.e., from five to six years of age) contributes to the progressive accumulation of predictive signal, reliable predictions sufficient to support individual selection decisions are not available before approximately seven years of age. From a practical perspective these findings suggest that trainers and breeders could begin systematic performance monitoring from approximately seven years of age. Focusing on cumulative performance patterns rather than individual results may support more efficient allocation of resources and tailored training programmes, while safeguarding horse welfare. While experienced practitioners may already apply similar reasoning intuitively, the present framework provides an objective and scalable tool that formalises these patterns quantitatively, which may be particularly valuable for large-scale screening and for stakeholders with less direct access to expert knowledge.

However, this model does not explicitly consider factors such as rider experience, training quality, environmental conditions, or individual temperament, all of which may influence performance outcomes. Future work could integrate additional biological, behavioural, and management data, assess model generalizability across horses from different countries, breeds and studbooks, and investigate the long-term welfare consequences of early competitive exposure. This study demonstrates the feasibility and value of machine learning for early talent identification in show jumping, providing a framework for evidence-based decision-making that could potentially support horse welfare indirectly by enabling earlier identification of horses unlikely to reach elite level, potentially allowing their redirection towards more suitable careers before excessive competitive demands are imposed.

## Supporting information

S1 File Formal definition of temporal windowing and feature extraction. This supplementary material provides the formal definition of the temporal windowing and feature extraction procedures described in the main text.
(DOCX)

S2 File Formal definition of evaluation metrics. This supplementary material provides the formal mathematical definition of the evaluation metrics used to assess model performance.
(DOCX)
