# Supplementary material for: Improving sport horse selection using machine learning: Early prediction of elite-level show jumping performance
Source: PLoS One. 2026 Sep 15;21(9):e0357799. doi: 10.1371/journal.pone.0357799 (PMC13577353; doi:10.1371/journal.pone.0357799)
Supplement: Supplementary file 1 [file pone.0357799.s001.docx]

**S1 File. Formal definition of temporal windowing and feature extraction**

This supplementary material provides the formal definition of the temporal windowing and feature extraction procedures described in the main text.

**Competition history representation**

For each horse i, the competition history was represented as an ordered sequence of competition events:

$$H_{i}=\{e_{i,1},e_{i,2},\ldots,e_{i,n_{i}}\}$$

where each event $e_{i,t}$ corresponds to a single competition and is described by the triplet:

$e_{i,t}$ = $\left( \text{age}_{i,t},\text{height}_{i,t},\text{clear}_{i,t} \right)$

With $\text{age}_{i,t}$ is the age of the horse at competition t; $\text{height}_{i,t}$is the obstacle height attempted and $\text{clear}_{i,t}$ is the binary indicator of clear-round performance (1 = clear, 0 = not clear).

**Temporal windowing procedure**

To generate fixed-length feature representations from variable-length competition histories, a growing-window approach was applied.

For each horse i, observation windows were defined as:

$$W_{i,L}=\left\{ e_{i,1,},\ldots,e_{i,L} \right\}$$

where L denotes the number of competitions included in the window.
The window length ranged from a minimum of $L_{min}$**= 5** competitions to the maximum number of available records for each horse (nᵢ).

**Feature extraction**

For each observation window $W_{i,L}$ summary statistics were computed to characterise the evolution of competition performance over time. The extracted features were grouped as follows.

*Performance indicators*

- Total number of clear rounds within the window
- Proportion of clear rounds within the window

*Age-related features*

- Minimum competition age
- Maximum competition age
- Age range (difference between maximum and minimum competition age)

*Obstacle-related features*

- Mean obstacle height attempted
- Maximum obstacle height attempted
- Standard deviation of obstacle height
- Highest obstacle height cleared
- Age at which the highest obstacle was cleared

**Feature matrix construction**

The temporal windowing procedure transformed each horse’s longitudinal competition history into a structured feature matrix, in which each row corresponded to a specific observation window from the horse’s career and each column represented a derived feature.

Each observation window was associated with a binary outcome label reflecting the horse’s final performance classification (elite-level performer vs. non-elite performer). This representation preserves the temporal progression of competition performance and supports the estimation of the probability of reaching elite-level performance across different stages of a horse’s competitive development.
