# Supplementary material for: Improving sport horse selection using machine learning: Early prediction of elite-level show jumping performance
Source: PLoS One. 2026 Sep 15;21(9):e0357799. doi: 10.1371/journal.pone.0357799 (PMC13577353; doi:10.1371/journal.pone.0357799)
Supplement: Supplementary file 2 [file pone.0357799.s002.docx]

**S2 File. Formal definition of evaluation metrics**

This supplementary material provides the formal mathematical definition of the evaluation metrics used to assess model performance.

**XGBoost evaluation metrics**

Model performance was evaluated on the external test folds of the nested cross-validation using both threshold-dependent and threshold-independent metrics. Let $\hat{p_{i}}$ denote the predicted probability for sample $i$, and $\hat{y_{i}}\in\{0,1\}$ the corresponding binary prediction obtained after applying the optimal classification threshold $\theta^{*}$. Model predictions were summarized in a confusion matrix, from which true positives (TP), false positives (FP), true negatives (TN) and false negatives (FN) were computed.

*Threshold-dependent metrics*

The following metrics were computed after applying the optimal classification threshold $\theta^{*}$:

- Accuracy (ACC):

$$ACC=\frac{TP+TN}{TP+FP+TN+FN}$$

- Sensitivity (SEN): true positive rate (also known as recall),

$$\text{SEN}=\frac{\text{TP}}{\text{TP}+\text{FN}}$$

- Specificity (SPEC): true negative rate,

$$\text{SPEC}=\frac{\text{TN}}{\text{TN}+\text{FP}}$$

Accuracy provides a global measure of correctness, while sensitivity and specificity quantify the model behaviour on each class. In the context of this study, sensitivity captures the ability to detect potential champion horses, whereas specificity measures the ability to correctly exclude non-champions. For each metric, the final reported score corresponds to the mean over the outer folds of the nested cross-validation.

*Threshold-independent metrics*

We employed two ranking-based metrics that evaluate classifier performance without requiring a predefined classification threshold:

- Area Under the ROC Curve (AUC): The AUC measures the probability that the classifier ranks a randomly chosen positive instance higher than a randomly chosen negative one. It is defined as the area under the Receiver Operating Characteristic (ROC) curve:

$$\text{AUC}=\int_{0}^{1} \text{SEN}\left( u \right) d\text{SPEC}\left( u \right)$$

- Average Precision (AP): The AP summarizes the precision--recall (PR) curve by computing the weighted mean of precision at each threshold, where the weight is the increase in recall:

$$\text{AP}=\sum_{k=1}^{n} \left( R_{k}-R_{k-1} \right) P_{k}$$

where $P_{k}$ and $R_{k}$ denote precision and recall at the $k-th$ threshold $\theta$, respectively. The recall is the sensitivity while the precision is defined as:

$$P\left( \theta\right)=\frac{\text{TP}\left( \theta\right)}{\text{TP}\left( \theta\right)+\text{FP}\left( \theta\right)}$$

**XGBoost hyperparameter search space**

Hyperparameter selection was performed using a randomised search (RandomizedSearchCV) with 40 iterations, run inside a 3-fold horse-grouped inner cross-validation scored by average precision. The search space is reported in table S1.

**Table S1.** XGBoost hyperparameter search space. Hyperparameter values considered during randomised search optimisation (RandomizedSearchCV, 40 iterations). Selection was performed using a 3-fold horse-grouped inner cross-validation scored by average precision.

| **Hyperparameter** | **Values** |
| --- | --- |
| n_estimators | 150, 300, 600, 900, 1200, 1500 |
| learning_rate | 0.02, 0.03, 0.05, 0.07, 0.1 |
| max_depth | 3, 4, 5, 6, 7 |
| min_child_weight | 1, 2, 4, 6, 8 |
| subsample | 0.6, 0.7, 0.8, 0.9, 1.0 |
| colsample_bytree | 0.6, 0.7, 0.8, 0.9, 1.0 |
| gamma | 0, 0.5, 1.0, 1.5, 2.0 |
| reg_lambda | 0.0, 0.5, 1.0, 2.0, 5.0, 10.0 |
| reg_alpha | 0.0, 0.5, 1.0, 2.0, 5.0 |
